# Diverse and Unexpected Roles of Human Monocytes/Macrophages in the Immune Response to Influenza Virus

**DOI:** 10.3390/v12040379

**Published:** 2020-03-31

**Authors:** Norbert J. Roberts

**Affiliations:** 1 Division of Infectious Diseases, Department of Internal Medicine, University of Texas Medical Branch at Galveston, Galveston, TX 77555, USA; norbert.roberts@nyulangone.org; 2 Division of Infectious Diseases and Immunology, Department of Medicine, New York University School of Medicine, New York, NY 10016, USA

**Keywords:** influenza virus, human monocytes, human macrophages, human lymphocytes, immune cell clusters, alveolar macrophages, lymphocyte activation, lymphocyte apoptosis

## Abstract

Human monocytes/macrophages play a central role in the immune response and defense of the host from influenza virus infection. They classically act as antigen-presenting cells for lymphocytes in the context of an immune cell cluster. In that setting, however, monocytes/macrophages exhibit additional, unexpected, roles. They are required for influenza virus infection of the lymphocytes in the cluster, and they are responsible for lymphocyte apoptosis via their synthesis and expression of the viral neuraminidase. Surprisingly, human alveolar macrophages, expected to be among the first cells to encounter the virus, are not susceptible to direct infection by a human influenza virus but can be infected when the virus is complexed with an antibody. Such monocyte/macrophage responses to influenza virus challenge should be considered part of a very complex but quite effective defense, since the common outcome is recovery of the host with development of immunity to the challenging strain of virus.

## 1. Introduction

Monocytes and macrophages are central to the development as well as expression of the human immune response and its defense against influenza A virus (IAV) infection. Such cells are resident in the respiratory tract [1,2,3,4], and additional monocytes/macrophages are rapidly and vigorously recruited to the respiratory tract upon virus challenge [3,4,5]. Those recruited cells are susceptible to infection by IAV, as are co-recruited lymphocytes [6,7]. The infection is abortive for both human leukocyte populations, with synthesis and expression of viral proteins such as the hemagglutinin, the neuraminidase, and the matrix protein, but without production of progeny virus [6,8,9,10]. 

Monocytes/macrophages participate in both the expression of innate immunity and in the development of the adaptive immune response that is responsible for recovery from IAV infection. In the former role, they can be formidable producers of interferon [4,11] and other cytokines [10], for example. In the latter role, they have long been recognized as important accessory cells for antigen presentation and activation of lymphocytes in response to the challenge [1,12,13]. In this brief review, unexpected roles of resident and recruited human monocytes/macrophages will be presented along with their classic role as antigen-presenting cells in response to human IAV infection.

## 2. Dichotomy in the Human Monocyte/Macrophage Antigen-Presenting Accessory Cell Function for Lymphocyte Responses to IAV Challenge

Early studies of human peripheral blood mononuclear cells (PBMC, consisting of monocytes/macrophages (PBM) and lymphocytes) documented that IAV infection resulted in a depression [14,15] or actual suppression [16] of standard measures of human cellular response, such as PBM-supported lymphocyte proliferation in response to stimulation by mitogens. Experiments using separated and recombined IAV-infected or sham-infected purified PBM and lymphocytes in cross-over assays indicated that the reduced proliferative response to phytohemagglutinin (PHA) was due to effects of the virus on the PBM [17]. Notably, however, aliquots of the IAV-exposed cells that responded poorly to mitogen stimulation were shown in concomitant assays to proliferate vigorously in response to IAV itself [16]. IAV-infected PBM actively supported IAV-specific lymphocyte activation and proliferation [16,18,19]. Furthermore, as noted above, the PBM actively responded to the IAV challenge in other ways, such as by the production of multiple cytokines, including interferon, interleukin (IL)-1, and tumor necrosis factor (TNF)α [10,11,20]. Thus, the accessory cell and other functions of PBM appeared to be focused on response to the challenging IAV [21].

It is reasonable to ask why PBM accessory cell function is reduced for responses to non-IAV stimulation. One possible host defense-related reason may be that proliferating cells show greater replication of IAV proteins [9], and it would benefit the host to limit such factories for the virus, having only lymphocytes responding specifically to the virus able to proliferate. Such observations may nonetheless be aligned with the widely held concept that a viral infection, such as due to IAV, can result in generally depressed cellular immunity [22].

Notably, the initiation of an immune response to IAV challenge involves the development of immune cell clusters between PBM and lymphocytes [23,24]. This challenge-induced structure provides the opportunity for PBM to act as accessory cells for the presentation of viral antigens to lymphocytes. However, as documented in the following sections, the immune cell cluster also allows certain unexpected PBM functions to occur during the immune response to IAV.

## 3. Human Monocytes/Macrophages are Directly Responsible for Lymphocyte Infection by IAV

Exposure of human PBMC to IAV resulted in the expression of viral antigens by both monocytes/macrophages and lymphocytes [7], resulting from new synthesis of the proteins by the cells [6]. If the cells were exposed to IAV as purified subpopulations of PBMC, monocytes/macrophages again synthesized and expressed viral antigens, but highly purified lymphocytes did not show any evidence of IAV synthesis or expression [6]. If the lymphocytes were exposed to IAV in co-culture with monocytes/macrophages and subsequently highly purified, both the monocytes/macrophages and lymphocytes synthesized and expressed viral antigens [6]. It was further shown that the presence of the monocytes/macrophages in co-culture was required, since cell-free supernatant fluids of either sham-infected or IAV-infected monocytes/macrophages did not facilitate lymphocyte infection. The requirement of monocytes/macrophages for lymphocyte infection was evident for strains from all three subtypes of human IAV (H1N1, H2N2, and H3N2) [6]. These data indicated that monocytes/macrophages are directly responsible and required for IAV infection of lymphocytes in the setting of the developing antiviral response.

Further studies demonstrated that the immune cell cluster plays an important role in IAV infection of human lymphocytes, even as that structure also serves to support development of the adaptive immune response [24]. Lymphocyte infection was decreased in a dose-dependent manner when anti-intercellular adhesion molecule-1 (ICAM-1) antibody was added to PBMC cultures to prevent clustering between monocytes/macrophages and lymphocytes. The studies also demonstrated that both peripheral blood lymphocytes and human alveolar lymphocytes required the presence of monocytes/macrophages for infection to occur [24]. Both CD4+ and CD8+ lymphocytes, and both resting and proliferating lymphocytes, became infected only when exposed to IAV in the presence of monocytes/macrophages.

## 4. Human Monocytes/Macrophages are Directly Responsible for Lymphocyte Apoptosis in the Setting of IAV Infection

After exposure to IAV in the presence of monocytes/macrophages, both infected and uninfected lymphocytes in the culture developed apoptosis [25]. Fas-FasL signaling played a major role in inducing apoptosis of the CD3+, CD4+, CD8+, and CD19+ subpopulations of lymphocytes after exposure to the virus. Since a large percentage of lymphocytes became apoptotic, but only a small percentage were infected, it was clear that direct effects from lymphocyte infection could not fully account for the findings. Furthermore, removal of monocytes/macrophages from the culture after exposure to IAV reduced the percentage of lymphocytes that underwent apoptosis [25].

Both depletion of monocytes/macrophages and depletion of cells expressing the IAV neuraminidase reduced the extent of lymphocyte apoptosis in the cultures [26]. Apoptosis was also reduced by the addition of anti-neuraminidase antibodies to the culture but not by the addition of anti-hemagglutinin antibodies. The extent of lymphocyte apoptosis varied with exposure to different strains of IAV, and the extent correlated directly with the production and expression of neuraminidase by infected monocytes/macrophages [26]. Thus, monocytes/macrophages appear to have a critical role in the development of lymphocyte apoptosis, even as they initiate an adaptive immune response and facilitate IAV infection of lymphocytes.

## 5. Unexpected Findings Regarding Interactions of Human Alveolar Macrophages (AM) with IAV

Monocytes/macrophages can serve as required accessory cells for antigen-stimulated and mitogen-stimulated proliferative responses, and they also play a central role in the regulation of immune responses [1]. Alveolar macrophages (AM) have been considered to be important cells for initiation and regulation of responses in the lung. AM may also regulate the growth and function of other non-leukocyte cells of the lung, such as fibroblasts [27]. Thus, it was expected that they would provide accessory cell support for lymphocyte responses to challenges such as streptococci or IAV. An extensive series of experiments using autologous monocytes, monocyte-derived macrophages, and AM from healthy human donors, co-cultured with lymphocytes, have raised questions about the regulatory role of alveolar macrophages for initiation of immune responses.

AM and PBM differed in their ability to enhance mitogen- and antigen-stimulated proliferative responses of purified autologous lymphocytes [1]. PBM supported, but AM did not support, lymphocyte proliferative responses to either streptococcal or (inactivated) IAV antigens. Such differences in accessory cell functions of the different monocytes/macrophages were not due to different kinetics of response, nor were they due to differences in the proportion of monocytes/macrophages required to support the lymphocyte responses [1]. Accessory cell support for lymphocyte proliferative responses to the antigen streptokinase-streptodornase (SK-SD) was even enhanced as peripheral blood monocytes matured to macrophages, but was absent when AM were added to the lymphocytes [28]. These data indicated that human AM have functional characteristics different from autologous PBM and raised the possibility that such differences may be important in both pulmonary immune homeostasis and in the pathogenesis of pulmonary disease, including that due to IAV challenge.

Mixing experiments were performed in which variable numbers of the two macrophages, PBM and AM, were co-cultured and assayed for support of SK-SD-stimulated proliferation of purified autologous lymphocytes [28]. Again, AM did not support the antigen-stimulated lymphocyte response, and even small numbers of AM suppressed SK-SD-stimulated lymphocyte proliferation supported by PBM in co-culture. The data thus indicated that diminished support exhibited by AM for antigen-stimulated lymphocyte responses was due at least in part to active suppression by the cells. Normal human AM clearly did not support antigen-stimulated proliferation [1,28]. Such regulation may relate to what is considered an important AM function in vivo, namely limiting inflammation in the lung [3,4].

IAV infection in vitro depresses accessory cell function of human PBM for lymphocyte proliferative responses, as described in Section 2, above. The effects of IAV infection of AM and autologous PBM were also compared by measuring the accessory support provided by these cells for phytohemagglutinin-induced proliferation of purified autologous lymphocytes [29]. Both PBM and AM can provide accessory cell support for lymphocyte responses to such a broad mitogenic stimulus. However, the cells again differ in this function and in the effect of IAV infection on the response. Accessory cell function of sham-infected AM was less effective for support of mitogen-stimulated lymphocytes proliferation than was that of sham-infected PBM. However, PBM support was significantly depressed by IAV infection. In contrast, this support function was not altered for the autologous IAV-exposed AM [29].

Differences between human autologous PBM and AM in the response to IAV, described above, warranted further studies regarding the susceptibility of the AM to IAV infection. PBM, as expected, bound and internalized IAV and synthesized viral RNA and proteins. In contrast, AM showed no evidence of IAV infection despite equivalent exposure [2]. The AM did become infected and synthesize IAV proteins if the cells were exposed to the virus in the presence of a sialidase inhibitor or an anti-IAV antibody or, in the case of exposure to a fluorescein isothiocyanate-labeled IAV, in the presence of anti–fluorescein isothiocyanate antibody. Thus, human AM are not susceptible to direct infection by IAV but can be infected when exposed to the virus complexed with an antibody [2].

## 6. Different Human Monocyte/Macrophage Responses to Pandemic and Avian IAV

All of the studies discussed above examined human monocyte/macrophage (including alveolar macrophages) interactions with seasonal human strains of IAV. The studies are consistent with the concept that alveolar macrophages are immunosuppressive in contrast to the more pro-inflammatory nature of monocytes/macrophages recruited to the lung in response to an infectious challenge [3]. In this section, we discuss studies of different human monocytes/macrophages exposed to pandemic or highly pathogenic avian IAV.

Although a human seasonal H1N1 virus caused an abortive infection in human alveolar macrophages, a highly pathogenic H5N1 virus from the 1997 Hong Kong outbreak replicated productively in the alveolar macrophages [30] and hyperinduced proinflammatory cytokines in human monocyte-derived macrophages [31]. Other studies examined differences between autologous human monocyte-derived macrophages and alveolar macrophages and found that the former cells produced virus and showed an excessive pro-inflammatory response when exposed to H5N1 virus. The H5N1-exposed alveolar macrophages showed lower virus production and lower pro-inflammatory mediator production [32]. Both H5N1/2004 and H5N1/1997 viruses were reported to replicate in human monocyte-derived macrophages, with multicycle infection [33]. In contrast, others have reported that human blood-derived macrophages were non-permissive for highly pathogenic H7N7 and H5N1 viruses [34]. There is also controversy in regard to the level of cytokine production by human monocytes/macrophages in response to pandemic H1N1 IAV infection. There are reports of greater cytokine production after exposure of human macrophages to avian H5N1 IAV compared to seasonal IAV exposure, with a pandemic H1N1 IAV inducing higher levels of several cytokines compared with seasonal IAV and with some, but not all, H5N1 strains of various clades [35]. The cytokine production profiles with exposure to the various IAV did not correlate with the viral replication levels in human macrophages. Another report indicated that human macrophages exposed to a pandemic H1N1 2009 strain of IAV induced a weak innate immune response [36]. The apparent differences in interactions of alveolar macrophages and peripheral blood-derived monocytes/macrophages with avian, pandemic, and seasonal IAV have been discussed previously, along with the identification of features of the studies that might result in discordant observations [37].

Overall, current evidence indicates that highly pathogenic strains of IAV, such as the avian H5N1 and the 1918 pandemic virus (which was an avian virus), can replicate in human monocytes/macrophages, including alveolar macrophages, but most other IAV infections of monocytes/macrophages are abortive, or nonproductive, and do not result in the release of infectious progeny virions. It is not evident that any published studies of pandemic or avian IAV infections have examined the interactions of human monocytes/macrophages and lymphocytes that are described in Section 2, Section 3, Section 4 and Section 5 above.

## 7. Animal Model Studies of Monocytes/Macrophages and IAV

Substantial insight regarding IAV infection pathogenesis has been derived from animal models of the infection. Animal models have allowed depletion of monocytes/macrophages, including alveolar macrophages, in order to demonstrate the importance of the cells during IAV infection of the host mouse [38,39] or ferret [40], the most commonly used animals. They have been especially useful in delineating the effects of infection with highly pathogenic avian IAV isolated from humans, including H5N1 viruses [41], as well as the avian virus that caused the 1918 human IAV pandemic [41]. They have been used to show that the depletion of alveolar macrophages was associated with a decrease in the expression of cytokines and chemokines that were otherwise observed to be increased with highly pathogenic IAV [41]. Murine studies have also delineated the adverse effects of monocyte/macrophage responses to IAV infection, such as the high numbers of macrophages accumulating in the lungs along with pro-inflammatory cytokines in response to highly pathogenic H5N1 IAV and the 1918 human pandemic virus (actually an avian virus) [42].

Mouse models have facilitated the examination of the effects of pregnancy on IAV-challenged alveolar macrophages [43] and on the monocytes/macrophages of juvenile mice versus adults, with persistent recruitment of monocytes to the lungs in the juvenile IAV infection [44], and allowed for an investigation of the role of specific host factors in the infection, such as protection from severe IAV infection by IL-36ɤ demonstrated using knockout mice and reconstitution with wild-type alveolar macrophages [45]. Recently, murine models with expanded tropism for human pathogens have been described [46], such as with implantation of human lung tissue into immunodeficient mice and challenge with another respiratory virus, namely respiratory syncytial virus.

Several caveats apply to murine studies as reflective of human infections. First, murine infections with human seasonal IAV strains, but not highly pathogenic avian strains such as H5N1 viruses or the 1918 human pandemic strain (an avian virus) or the H1N1 2009 human pandemic strain, require prior adaption to the species that are not naturally susceptible [47,48]. Second, mouse genetic strains can differ with regard to the effects of IAV infection on monocytes/macrophages such as alveolar macrophages [49]. Third, there are documented differences between mouse and human immunology [50].

## 8. Conclusions

Human monocytes/macrophages exhibit several unexpected characteristics relating to IAV infection, as described above: (a) an immunofocusing of monocyte/macrophage function, whereby non-IAV-directed immune responses are depressed or suppressed concomitant with the development of active IAV-specific responses; (b) responsibility of the monocytes/macrophages, in the setting of the immune cell cluster of the developing antiviral response, not only for lymphocyte activation, but also for lymphocyte infection by IAV, as well as lymphocyte apoptosis arising from monocyte/macrophage expression of IAV neuraminidase; and (c) a surprising resistance of AM to direct infection by a human IAV.

A recent report of sequential experimental H1N1pdm09 infection of volunteers indicated the possibility of re-infection with the same strain of IAV [51]. In general, however, it is important to recognize that, in the great majority of individuals challenged by a human IAV, the result of combined monocyte/macrophage and lymphocyte responses is recovery and the establishment of an important degree of immune defense against severe disease with re-exposure to the challenging strain of virus. Thus, the unexpected characteristics described above should not be considered clearly adverse aspects of monocyte/macrophage responses to IAV challenge but as part of a very complex but quite effective defense.
